# Bibliometric analysis of fibroblast growth factor 21 research over the period 2000 to 2021

**DOI:** 10.3389/fphar.2022.1011008

**Published:** 2022-09-27

**Authors:** Qin-Ying She, Li-Juan Li, Ming-Hong Liu, Ru-Yu Tan, Yi-Wen Zhong, Jing-Fu Bao, Jie-Dong Xie

**Affiliations:** ^1^ Department of Nephrology, The Fifth Affiliated Hospital, Southern Medical University, Guangzhou, China; ^2^ State Key Laboratory of Organ Failure Research, National Clinical Research Center for Kidney Disease, Nanfang Hospital, Southern Medical University, Guangzhou, China; ^3^ Guangdong Provincial Key Laboratory of Renal Failure Research, Guangzhou Regenerative Medicine and Health Guangdong Laboratory, Guangzhou, China

**Keywords:** bibliometric analysis, fibroblast growth factor 21, Histcite, VOSviewer, Citespace

## Abstract

**Background:** Fibroblast growth factor 21 (FGF-21) is an evolutionarily conserved protein that plays multiple roles in metabolic regulation. Over the past two decades, numerous studies have deepened our understanding of its various functions and its pharmacological value. Nevertheless, most clinical trials have not achieved the desired results, which raises issues regarding its clinical value. In this bibliometric analysis, we evaluated the state of FGF-21 research over the last 20 years and identified important topics, achievements, and potential future directions.

**Methods:** Publications related to FGF-21 were collected from the Web of Science Core Collection-Science Citation Index Expanded. HistCite, VOSviewer, and CiteSpace were used for bibliometric analysis and visualization, including the analysis of annual publications, leading countries, active institutions and authors, core journals, co-cited references, and keywords.

**Results:** Altogether, 2,490 publications related to FGF-21 were obtained. A total of 12,872 authors from 2,628 institutions in 77 countries or regions reported studies on FGF-21. The United States of America was the most influential country in FGF-21 research. Alexei Kharitonenkov, Steven A. Kliewer, and David J. Mangelsdorf were the most influential scholars, and endocrinology journals had a core status in the field. The physiological roles, clinical translation, and FGF-21-based drug development were the main topics of research, and future studies may concentrate on the central effects of FGF-21, FGF-21-based drug development, and the effects of FGF-21 on non-metabolic diseases.

**Conclusion:** The peripheral metabolic effects of FGF-21, FGF-21-based drug development, and translational research on metabolic diseases are the three major topics in FGF-21 research, whereas the central metabolic effects of FGF-21 and the effects of FGF-21 on metabolic diseases are the emerging trends and may become the following hot topics in FGF-21 research.

## Introduction

Fibroblast growth factor (FGF) is a large protein family with 22 members, which can function as paracrine or endocrine factors related to many biological processes, including embryogenesis, wound healing, angiogenesis, and metabolism (Beenken and Mohammadi, 2009). Based on sequence homology and phylogeny, the FGF family can be divided into several subfamilies, among which the endocrine FGF-19 subfamily consists of three FGFs, namely, FGF-19 (FGF-15 in rodents), FGF-21, and FGF-23 (Beenken and Mohammadi, 2009). As a second member of the FGF-19 subfamily, FGF-21 was identified in 2000 (Nishimura et al., 2000); however, its exact function remained unknown for five years. In 2005, Kharitonenkov et al. reported the glucose- and lipid-lowering effects of FGF-21 (Kharitonenkov et al., 2005), which was the first finding regarding its metabolic functions. Subsequently, β-klotho was revealed to be indispensable for FGF-21-induced fibroblast growth factor receptor (FGFR) activation and metabolic improvements (Ogawa et al., 2007), and the expression of β-Klotho is limited to metabolism-related tissues (She et al., 2022). These results confirm the metabolic functions of FGF-21. Notably, FGF-21 can significantly mitigate metabolic disorders without inducing hypoglycemia or mitogenesis (Kharitonenkov et al., 2005), further implying its significant potential in clinical applications.

Since these discoveries, an increasing number of studies have shown that it can act on adipose tissue, pancreas, liver, muscle, and hypothalamus to integrally regulate glucometabolism and lipometabolism (She et al., 2022). In addition, several FGF-21-based bioengineered drugs were developed, taking into consideration the short half-life of native FGF-21 *in vivo* (Kharitonenkov et al., 2007; Huang et al., 2013), such as LY2405319 (Kharitonenkov et al., 2013), PF-05231023 (Huang et al., 2013), bFK1 (Kolumam et al., 2015), AKR-001 (Stanislaus et al., 2017), and BMS-986036 (Charles et al., 2019). The glucose-lowering effects of these drugs were obvious in diabetic/obese rodents (Huang et al., 2013; Kharitonenkov et al., 2013; Kolumam et al., 2015; Weng et al., 2015) and diabetic non-human primates (Adams et al., 2013a; Thompson et al., 2016; Baruch et al., 2020), whereas such effects were not observed in most of the clinical trials (Gaich et al., 2013; Talukdar et al., 2016; Kim et al., 2017; Baruch et al., 2020), indicating that the precise functions of FGF-21 remain poorly understood.

Bibliometrics was first introduced by Pritchard (1969) in 1969, and it is used as a quantitative and statistical method for analyzing large volumes of scientific data. As an analytic model, bibliometric analysis can quickly reveal the knowledge network, evolution of the research topic, and emerging trends of a specific research field by analyzing relevant literature (Chen and Song, 2019). In recent years, bibliometrics has been applied to present the evolution of specific research fields, such as information science (He et al., 2017), decision science (Lin et al., 2020; Luo and Lin, 2021; Yu et al., 2021; Zhang and Lin, 2022), environmental science (Chen et al., 2022), artificial intelligence (Wamba et al., 2021), sociology (Foroudi et al., 2020), and medicine (Rubagumya et al., 2022). On the other hand, bibliometric analysis has been used to analyze certain journals to better understand their scope, such as the *Journal of Nursing Management* (Yanbing et al., 2020), *Journal of Business Research* (Dhontu et al., 2020), and *Pharmacological Research* (Hassan et al., 2021). As a rapidly growing field, biomedical research is especially well-adapted for bibliometric analysis. For example, Zhang et al. analyzed diabetic kidney disease-induced renal fibrosis research through bibliometric analysis and revealed the top contributing countries and authors and the most popular journals in this field. Most importantly, they also demonstrated main research topics, including “microRNAs”, “bone morphogenetic protein”, “hypertrophy”, “glomerulosclerosis”, and “diabetic kidney disease”, and identified that “microRNAs” is a hot topic in recent works (Zhang et al., 2022). Thus, bibliometric analysis can be performed to provide an in-depth evaluation of the development of the research concerning FGF-21, and this can guide us in future work. To our knowledge, bibliometric analysis of FGF-21 research is lacking, and our aim is to analyze publications on FGF-21 to evaluate the current status and potential future directions.

## Methods

### Search strategy in Web of Science Core Collection

Although PubMed contains more biomedicine-related publications than Web of Science Core Collection (WoSCC), the latter has a better citation network, which is important for co-citation analysis. WoSCC includes the most influential publications on FGF-21 research, which avoids the omission of important research studies to a large extent. Furthermore, HistCite, VOSviewer, and CiteSpace are the most suitable for analyzing WoSCC-derived datasets. Hence, we conducted a literature search mainly on the Science Citation Index Expanded (SCIE) on the WoSCC. Topic search is a search model of words in titles, abstracts, author keywords, and keywords plus, and we chose a topic search to precisely obtain the dataset of FGF-21. The search formula was set as follows: Topic Search (TS) = (“fibroblast growth factor 21” OR “FGF21” OR “FGF 21”). The publication years were restricted to the period between 2000 and 2021, and the document types were “articles” or “reviews”. The article language was set as English. Then, the publication number, publication titles, authors, affiliations, countries, keywords, published journals, publication years, references, and citations were collected for analysis. We finally obtained 2,492 publications, and two retracted publications were removed (Supplementary Table S1). To avoid deviations from WoSCC updates, all of the abovementioned operations were completed within one day (25 June 2022).

### Bibliometric analysis

HistCite (version 12.03.17) (Garfield et al., 2006) was used to analyze the publication number, total global citation score (TGCS), and total local citation score (TLCS) for each publication year, active countries, active institutions, active authors, and core journals. TGCS is the number of citations in WoSCC, and TLCS is the number of citations in the current dataset.

Co-citation and co-occurrence networks were completed using VOSviewer (version 1.6.18) (van Eck and Waltman, 2010). Collaborations between countries, institutions, and authors and mutual citations between journals were analyzed using co-citation networks. Keyword occurrences in various publications were analyzed using co-occurrence networks. The minimum number of documents analyzed for co-citation and co-occurrence is presented in the corresponding sections. The same color indicates close cooperation, and the total link strength of an item reflects the degree of cooperation with others. In addition, the size of the nodes in the networks represents the total link strength of the items, and the thickness of the connecting line in the networks indicates the strength of the links. Scimago Graphica was also involved in international collaboration analysis.

CiteSpace (version 5.8.R3) (Chen, 2004) was used to analyze the knowledge domain in FGF-21, including dual-map overlay, cluster, centrality, timeline view, reference burst analysis, and keyword burst analysis. Time slicing was set from 2000 JAN to 2021 DEC, with two years per slice. The node type was set as “References” or “Keywords”, and the selection criteria were set as the top 50 levels of most cited or occurred items from each slice. Modularity Q and mean silhouette were used to evaluate the reliability of clustering; Q > 0.3 and mean silhouette >0.5 indicated enough clustering structure and convincing clustering results, respectively. Moreover, CiteSpace was used to analyze publications from the last five years (2017–2021). Time slicing was set from 2017 JAN to 2021 DEC, with one year per slice. The node type was set as “References”, and the selection criteria were set as the top 50 levels of most cited or occurred items from each slice.

## Results

### An overview of FGF-21-related publications

**FIGURE 1:**
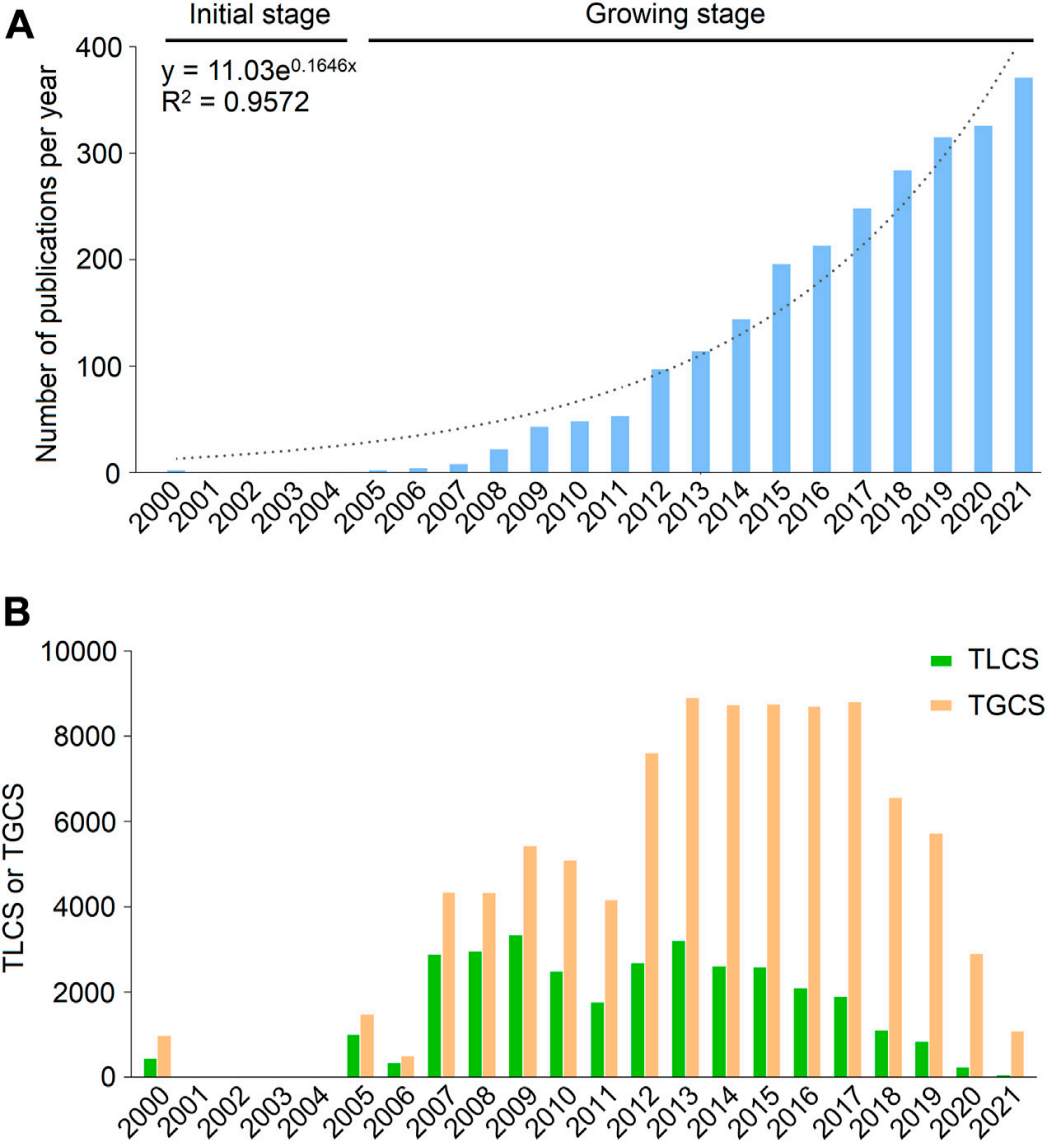
Publication outputs and citations on FGF-21 in the past 22 years. **(A)** Global annual production trends. Blue bars represent the number of publications related to FGF-21 per year, and the dotted line represents the trend-fitted curve. The fitting equation and the correlation coefficients (R^2^) are presented. **(B)** Annual TGCS and TLCS of publications.

A total of 2,490 publications related to FGF-21 were obtained, including 2,172 articles and 318 reviews (Supplementary Table S2). Global publications in the field demonstrated an overt growth trend, from two publications in 2000 to 371 publications in 2021 (Figure 1A). This can be divided into two stages according to the annual publications: the initial stage (2000–2004) and the growing stage (2005–2021). Initially, only two articles were published, one of which was published by Nishimura et al., who identified the existence of FGF-21 in mammals (cited 582 times) (Nishimura et al., 2000). In 2005, Kharitonenkov et al. first demonstrated that FGF-21 can act as a metabolic regulator to normalize metabolic disorders without inducing hypoglycemia and neoplasm (cited 1452 times) (Kharitonenkov et al., 2005), which resulted in a significant increase in FGF-21-related publications.

Publications concerning FGF-21 were cited 93,982 times on WoSCC, with an average of 37.74 times per article. Corresponding to publication number, the TGCS and TLCS of publications were relatively low in the initial stage; however, the TGCS exhibited a gradual increase from 2005 to 2013 and peaked in 2013 (8898 citations). Since 2014, the TGCS and TLCS have been relatively stable (Figure 1B), further indicating that FGF-21 research has remained a hot topic in recent years.

### Countries leading in FGF-21 research

**FIGURE 2:**
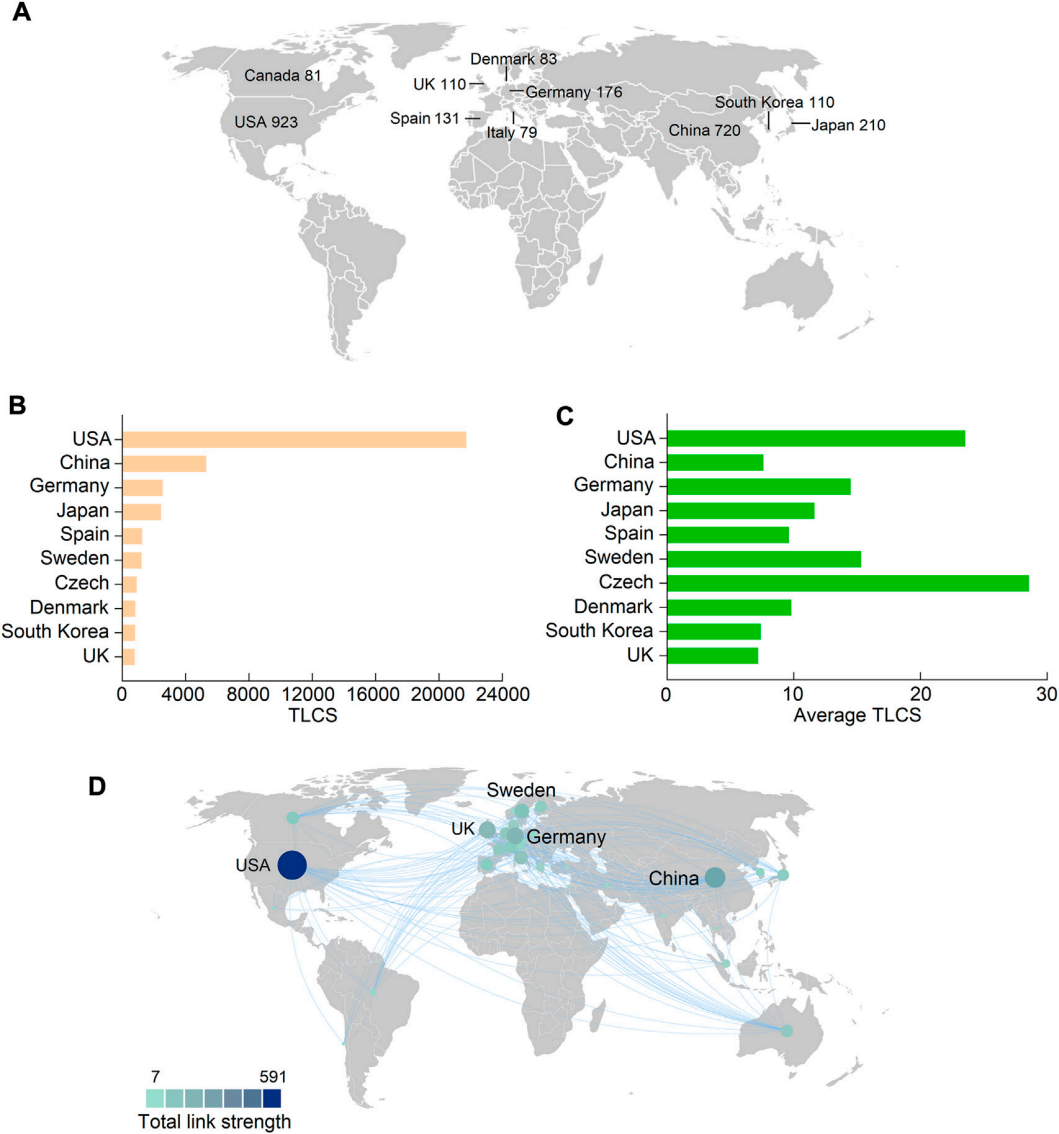
Leading countries in the field of FGF-21 research. **(A)** The top ten productive countries concerning FGF-21. **(B and C)** TLCS and average TLCS of the top ten countries with the highest TLCS. **(D)** The international collaboration among different countries. Each country is represented as a node, and each line means a co-authorship relationship. The node size is proportional to the collaboration link strength.

A total of 77 countries and regions contributed to FGF-21 research, and the United States of America (United States) contributed the largest number of publications (923), followed by China (720), Japan (210), Germany (176), and Spain (131) (Figure 2A, Supplementary Table S3). Correspondingly, studies from the United States had the highest number of TGCS (54,087 citations), followed by those from China (15,823), Japan (8,595), Germany (7,612), and Spain (5,610); the rest were all less than 5,000 citations (Supplementary Table S3). As mentioned earlier, the TLCS is the number of citations in the current dataset, and it can better reflect the influence of publications in the field of FGF-21 research. Compared to TGCS, the first five countries with the highest TLCS were the United States (21,715 citations), China (5,328), Germany (2,550), Japan (2,443), and Spain (1,258) (Figure 2B), further indicating their dominant roles in FGF-21 research. Notably, although China had a high publication number, TGCS, and TLCS (Figure 2A, Figure 2B, and Table 1), its average TLCS and TGCS were relatively low (Figure 2C, Table 1, and Supplementary Table S3), implying a minor influence of these studies.

**TABLE 1:** Top ten countries with the highest TLCS.

Rank	Country	Publications	TLCS	TGCS	Average TLCS	Betweenness centrality
1	United States	923	21715	54087	23.53	0.33
2	China	720	5328	15823	7.40	0.16
3	Germany	176	2550	7612	14.49	0.09
4	Japan	210	2443	8595	11.63	0.04
5	Spain	131	1258	5610	9.60	0.08
6	Sweden	79	1210	3812	15.32	0.07
7	Czech Republic	32	914	1624	28.56	0.17
8	Denmark	83	813	2298	9.80	0.01
9	South Korea	110	812	2885	7.38	0.06
10	United Kingdom	110	790	3523	7.18	0.16

There were 30 countries with more than ten publications that were included in the co-authorship analysis. The highest total link strength was observed in the United States (total link strength = 591 times) (Figure 2D). Betweenness centrality is an indicator of a node’s impact on a network; a node with higher betweenness centrality has a greater influence on the transfer of information through the network. Corresponding to the total link strength, the betweenness centrality of the United States, in collaboration, was the highest (Table 1), further indicating its critical role in international cooperation. In this largest cooperative network led by the United States, China (294), Germany (200), the United Kingdom (United Kingdom) (190), and Sweden (149) were in key positions (Figure 2D).

### Active institutes and authors in FGF-21 research

Altogether, 12,872 authors from 2,628 institutions have published reports regarding FGF-21. The top ten productive institutions were mostly in China (three institutions) and the United States (three institutions). Wenzhou Medical University published the most articles (106 publications), followed by the University of Barcelona (66), the University of Hong Kong (59), Harvard Medical School (57), and the University of Copenhagen (57) (Supplementary Table S4). However, in the top ten institutions with the highest TLCS, there was only one Chinese institution (the University of Hong Kong), and the rest were almost entirely from the United States, except Kyoto University (Table 2).

**TABLE 2:** Top ten institutions with the highest TLCS.

Rank	Institution	Publications	TLCS	TGCS	Average TLCS
1	University of Texas, Southwestern Medical Center at Dallas	32	3523	6285	110.09
2	Lilly Research Laboratories	28	2762	4241	98.64
3	Eli Lilly and Company	30	2620	4203	87.33
4	New York University	25	2545	4392	101.80
5	The University of Hong Kong	59	2506	4360	42.47
6	Harvard University	46	2482	5485	53.96
7	Beth Israel Deaconess Medical Center	20	1836	3266	91.80
8	Amgen Incorporation	40	1673	3025	41.83
9	Kyoto University	42	1486	3769	35.38
10	Lilly Cooperate Center	22	1457	2573	66.23

**FIGURE 3:**
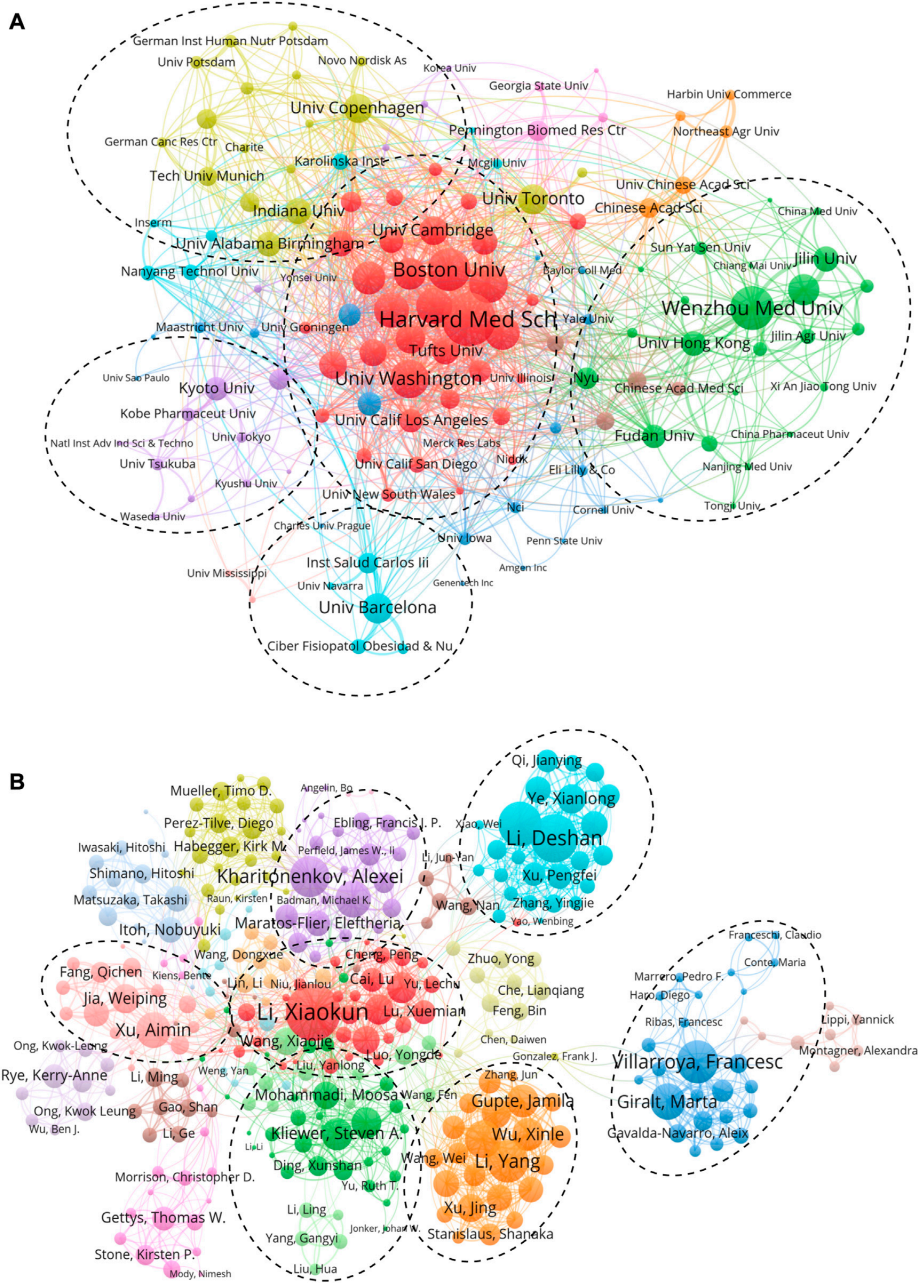
Active institutes and authors analysis. **(A)** Clustering of collaboration among institutes. **(B)** Clustering of collaboration among authors. In these maps, each institute or author is represented as a node, and each line means a co-authorship relationship. The node size is proportional to the collaboration link strength, and the line thickness indicates the collaboration link strength. In addition, the node color reflects the cluster to which it belongs.

We further analyzed the co-authorship of 141 institutions with more than five publications, while the institutions that were not connected were removed, leaving behind 138 institutions. Harvard Medical School showed the most frequent collaboration with other institutions (total link strength = 147 times) (Figure 3A), followed by Wenzhou Medical University (113), Boston University (110), University of Washington (95), and Brigham and Women’s Hospital (88). Cooperation among the institutions could be divided into five clusters, and cooperation led by Harvard Medical School showed the most complex network (Figure 3A).

The top ten most productive authors were Xiaokun Li of Wenzhou Medical University (68 publications), Alexei Kharitonenkov of AK Biotechnologies LLC (64), Francesc Villarroya of Universitat de Barcelona (49), Deshan Li of Northeast Agricultural University (48), Aimin Xu of The University of Hong Kong (44), Andrew C. Adams of Lilly Research Laboratories (39), Marta Giralt of Universitat de Barcelona (34), Nobuyuki Itoh of Kyoto University (33), Guiping Ren of Northeast Agricultural University (32), and Weiping Jia of Shanghai Jiaotong University (29). Notably, half of the authors were from China (Supplementary Table S5). Nevertheless, almost all the top ten authors with the highest TLCS were from the United States, except for Aimin Xu (Table 3).

**TABLE 3:** Top ten authors with the highest TLCS.

Rank	Name	Publications	TLCS	TGCS	Institutions
1	Alexei Kharitonenkov	64	6990	11085	AK Biotechnology LLC
2	Steven A. Kliewer	27	3777	6577	University of Texas Southwestern Medical Center at Dallas
3	David J. Mangelsdorf	25	3483	6020	University of Texas Southwestern Medical Center at Dallas
4	Eleftheria Maratos-Flier	28	2989	5273	Novartis Institutes for BioMedical Research
5	Moosa Mohammadi	19	2528	4261	New York University
6	Aimin Xu	44	2417	4078	The University of Hong Kong
7	Regina Goetz	10	2233	3715	New York University
8	Jeffrey S. Flier	16	2185	3755	Harvard Medical School
9	Xunshan Ding	10	2154	3402	Southwestern Medical Center at Dallas
10	Holly A. Bina	10	2018	2789	Lilly Research Laboratories

A total of 379 authors who had more than ten publications were included in the co-author analysis, and 52 authors that were not connected were excluded. These authors were divided into the seven largest collaboration networks, which were constructed by Xiaokun Li (total link strength = 231 times), Deshan Li (182), Francesc Villarroya (151), Yang Li (147), Alexei Kharitonenkov (140), Aimin Xu (115), and Steven A. Kliewer (98). The collaboration networks of Xiaokun Li, Deshan Li, Alexei Kharitonenkov, and Yang Li mainly focused on drug discovery and translational research based on FGF-21, and the collaboration network of Aimin Xu focused on the clinical and translational aspects of FGF-21, while the networks of Steven A. Kliewer’s and Francesc Villarroya concentrated on the physiological mechanisms of FGF-21. These collaborative networks represent the main topics in the FGF-21 research field. Interestingly, Francesc Villarroya’s network demonstrated less collaboration with the other groups (Figure 3B). Collectively, the results of active institutions and authors also imply the leading role of the United States in the field of FGF-21.

### Core journals in FGF-21 research

FGF-21-related studies were published in 695 journals. The top ten journals with the highest number of publications are shown in Supplementary Table S6, and approximately 20.64% of all publications were published in these journals. Interestingly, although PLoS One, Scientific Reports, and the International Journal of Molecular Sciences published a larger number of articles, these articles were less cited by FGF-21-related articles. Table 4 shows the top ten journals with the highest TLCS, which better reflects the influence of these journals on FGF-21-related topics. Approximately 52.20% of the total TLCS occurred in the top ten journals with the highest TLCS, indicating their significant influence. Among these journals, six were endocrinology journals, namely, Cell Metabolism (TLCS: 4428), Diabetes (2918), Endocrinology (2641), Journal of Clinical Endocrinology & Metabolism (1216), Molecular Metabolism (888), and Clinical Endocrinology (603). Significantly, although the Journal of Clinical Investigation only published nine FGF-21 research articles, the TLCS of these articles ranked fourth (Table 4). This was mainly attributed to the article “*FGF-21 as a novel metabolic regulator*”, which first identified the biological functions of FGF-21 (Kharitonenkov et al., 2005).

**TABLE 4:** Top ten journals with the highest TLCS.

Rank	Journal	Counts	Impact factor (2021)	TLCS	TGCS	H index
1	Cell Metabolism	43	31.373	4428	9002	39
2	Diabetes	43	9.337	2918	5001	29
3	Endocrinology	51	5.051	2641	4394	30
4	Journal of Clinical Investigation	9	19.456	1432	2299	8
5	Proceedings of the National Academy of Sciences of the United States of America	22	12.779	1276	2690	17
6	Journal of Biological Chemistry	36	5.486	1216	2779	26
7	Journal of Clinical Endocrinology & Metabolism	40	6.134	907	1539	25
8	Molecular Metabolism	53	8.568	888	1889	23
9	Clinical Endocrinology	16	3.523	603	868	12
10	FEBS Letters	7	3.864	595	859	6

**FIGURE 4:**
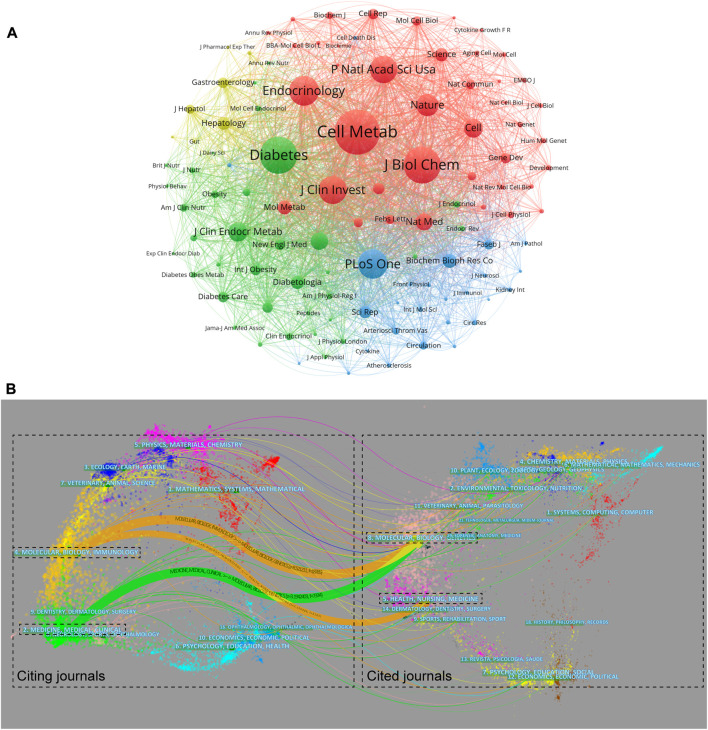
Core journals in the field of FGF-21 research. **(A)** Clustering of co-citation among journals. Each node represents a journal, and each line means a co-cited relationship. The node size is proportional to the co-cited link strength, and the node color reflects the cluster to which it belongs. **(B)** The dual-map overlay of articles citing FGF-21 research. The left and right sides are the citing and cited journals, respectively, and the line path represents the citation relationship.

A total of 109 journals that were cited more than 200 times were included in the co-cited analysis. Cell Metabolism (total link strength = 415,641 times), Diabetes (321,286), Journal of Biological Chemistry (318,210), Endocrinology (245,442), and Journal of the Clinical Investigation (226,705) had the most co-citations with other journals (Figure 4A), suggesting their important status in FGF-21 research. The dual-map overlay showed three main citation pathways. As the cited journals provide the knowledge base of the citing journals, such citation pathways showed that the investigations on FGF-21 mainly concentrated on “molecular, biology, immunology” and “medicine, medical, clinical”, and these works were mainly based on studies on “molecular, biology, genetics” and “health, nursing, medicine” (Figure 4B).

### Co-cited publications in FGF-21-related field

The top 20 publications with the highest TLCS are listed in Table 5. These publications were all research articles, most of which were physiological or translational research. Among these publications, four were clinical investigations and one was related to drug research/development, indicating that FGF-21-based drug discovery and the clinical value of FGF-21 are also important.

**TABLE 5:** Top ten publications with the highest TLCS.

Rank	First author	Journal	Year	Category	TLCS	TGCS
1	Kharitonenkov Alexei	Journal of Clinical Investigation	2005	Translational research	992	1452
2	Takeshi Inagaki	Cell Metabolism	2007	Physiological research	689	1084
3	Michael K. Badman	Cell Metabolism	2007	Physiological research	684	1087
4	Jing Xu	Diabetes	2009	Translational research	594	812
5	Tamer Coskun	Endocrinology	2008	Translational research	568	755
6	Xinmei Zhang	Diabetes	2008	Clinical research	468	634
7	Kharitonenkov Alexei	Endocrinology	2012	Translational research	451	578
8	Ffolliott M. Fisher	Genes & Development	2012	Physiological research	427	1006
9	Tetsuya Nishimura	Biochimica et Biophysica Acta	2000	Physiological research	417	582
10	Ffolliott M. Fisher	Diabetes	2010	Translational research	376	506
11	Gregory Gaich	Cell Metabolism	2013	Drug discovery and clinical research	375	579
12	Matthew J. Potthoff	Proceedings of the National Academy of Sciences of the United States of America	2009	Physiological research	345	486
13	Yasushi Ogawa	Proceedings of the National Academy of Sciences of the United States of America	2007	Physiological research	308	438
14	Hiroshi Kurosu	Journal of Biological Chemistry	2007	Physiological research	294	544
15	Jody Dushay	Gastroenterology	2010	Clinical research	290	403
16	Cecilia Gälman	Cell Metabolism	2008	Clinical research	285	377
17	Wolf Wente	Diabetes	2006	Translational research	265	365
18	Zhuofeng Lin	Cell Metabolism	2013	Translational research	257	402
19	Kathleen R. Markan	Diabetes	2014	Physiological research	247	334
20	Klementina Fon Tacer	Molecular Endocrinology	2010	Physiological research	243	453

**FIGURE 5:**
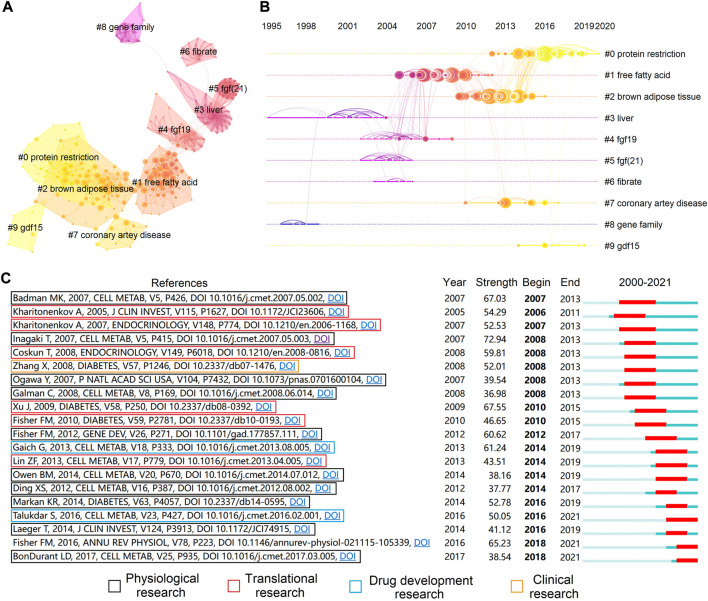
Co-cited references analysis. **(A)** Cluster analysis of co-cited references. Each line indicates a co-cited relationship. **(B)** Timeline view of the ten clusters. The position of the node on the horizontal axis indicates the time point of the first appearance, and each line represents a co-cited relationship. The size of each node is proportional to the number of citations. **(C)** The top twenty references with the highest citation bursts.

Analysis of keyword clustering demonstrated ten rational clusters, whereas clusters without co-cited these largest clusters were excluded (Figure 5A and Table 6). The modularity Q and mean silhouette value of clustering were 0.8106 and 0.9502, respectively, indicating a reliable structure and results of clustering. We then created a visualized timeline for these clusters to identify the involvement of FGF-21-related research topics (Figure 5B). The identification and classification of liver-expressed FGF-21 were early topics in FGF-21 research (Nishimura et al., 2000) (Cluster ID #3 and ID #8). As a member of the FGF-19 subfamily (Beenken and Mohammadi, 2009), the metabolic functions of FGF-21 are similar to those of FGF-19 (Dolegowska et al., 2019), and β-klotho is required for their metabolic effects (Kurosu et al., 2007; Ogawa et al., 2007; Ding et al., 2012). Hence, “fgf19” (Cluster ID #4) and “fgf (21)” (Cluster ID #5) were co-cited in subsequent studies, and the molecular mechanisms of their metabolic effects were hot topics during this period. The mechanism by which FGF-21 exerts its metabolic effects is a major concern. Inagaki et al. found that *Fgf21*-transgenic mice exhibited increased free fatty acid levels (Inagaki et al., 2007) and that FGF-21 induces lipolysis. Nevertheless, Arner et al. demonstrated that FGF-21 attenuates lipolysis *in vitro* and further suggested that this is a possible link that mediates FGF-21-induced insulin sensibilization (Arner et al., 2008). In addition, FGF-21 was found to stimulate the browning of white adipose tissues (Fisher et al., 2012), which may promote glucose uptake (Xu et al., 2009a; Ding et al., 2012; Markan et al., 2014; BonDurant et al., 2017). These studies placed “free fatty acid” and “brown adipose tissue” into the central stage (Cluster ID #1 and ID #2). The metabolic effects associated with FGF-21 levels in several metabolism-related diseases, such as coronary artery disease (Shen et al., 2013), may provide diagnostic value (Cluster ID #7). Laeger et al. demonstrated that protein restriction promotes FGF-21 expression and mediates metabolic alterations of protein restriction (Laeger et al., 2014). Since protein restriction was correlated with metabolic and aging-related diseases (Wang et al., 2022), the role of FGF-21 in protein restriction and metabolic diseases/aging has been an important topic in recent years (Cluster ID #0).

**TABLE 6:** Top ten clusters of co-cited references with the highest K value.

Cluster ID	Size	Silhouette	Mean year	Top term	Log (likelihood ratio, p level)
#0	49	0.935	2016	Protein restriction	17.67, 1.0E-4
#1	48	0.973	2008	Free fatty acid	8.91, 0.001
#2	48	0.903	2012	Brown adipose tissue	9.33, 0.005
#3	26	0.935	2000	Liver	4.83, 0.005
#4	25	0.950	2005	Fgf19	13.64, 0.001
#5	17	0.990	2004	Fgf (21)	9.05, 0.005
#6	13	1	2004	Fibrate	10.48, 0.005
#7	11	0.975	2013	Coronary artery disease	19.31, 1.0E-4
#8	11	1	1997	Gene family	12.36, 0.001
#9	8	0.974	2017	Gdf15	20.20, 1.0E-4

The term “citation burst” refers to references that were frequently cited over a period of time, and burst detection can reflect the hotspots in a certain period based on the topic of references. We conducted citation burst detection and identified the top 20 references with the strongest citation burst (Figure 5C). Almost all publications were research articles, and there was only one review (Fisher and Maratos-Flier, 2016). Articles with high impact from 2005 to 2012 mostly concentrated on the physiological roles (Badman et al., 2007; Inagaki et al., 2007; Ogawa et al., 2007; Gälman et al., 2008; Ding et al., 2012; Fisher et al., 2012) and preclinical applications of FGF-21 (Kharitonenkov et al., 2005; Kharitonenkov et al., 2007; Coskun et al., 2008; Xu et al., 2009a; Fisher et al., 2010). Based on these studies, FGF-21 was considered an ideal medicine for the treatment of metabolic diseases. However, native FGF-21 is unsuitable for clinical application because of its short half-life (Kharitonenkov et al., 2007; Huang et al., 2013), susceptibility to proteolytic inactivation (Coppage et al., 2016; Dunshee et al., 2016; Zhen et al., 2016), and improper aggregation under physiological conditions (Kharitonenkov et al., 2013). In 2013, the first FGF-21 analogue, LY2405319, was designed to overcome the defect of native FGF-21 (Kharitonenkov et al., 2013), and this promoted the design of other analogues, such as PF-05231023 (Huang et al., 2013). Nevertheless, although LY2405319 and PF-05231023 demonstrated satisfactory glucose- and lipid-lowering effects in rodents and nonhuman primates (Adams et al., 2013a; Huang et al., 2013; Kharitonenkov et al., 2013; Weng et al., 2015), the glucose-lowering effect was absent in obese human with diabetes (Gaich et al., 2013; Talukdar et al., 2016; Kim et al., 2017). Such inconsistencies indicated an inadequate understanding of FGF-21 and motivated scholars to further investigate the metabolic role of FGF-21 (Bookout et al., 2013; Laeger et al., 2014; Markan et al., 2014; Owen et al., 2014; BonDurant et al., 2017). Notably, the effects of FGF-21 on the central nervous system came into focus after 2013 (Owen et al., 2014).

### Keywords analysis

**FIGURE 6:**
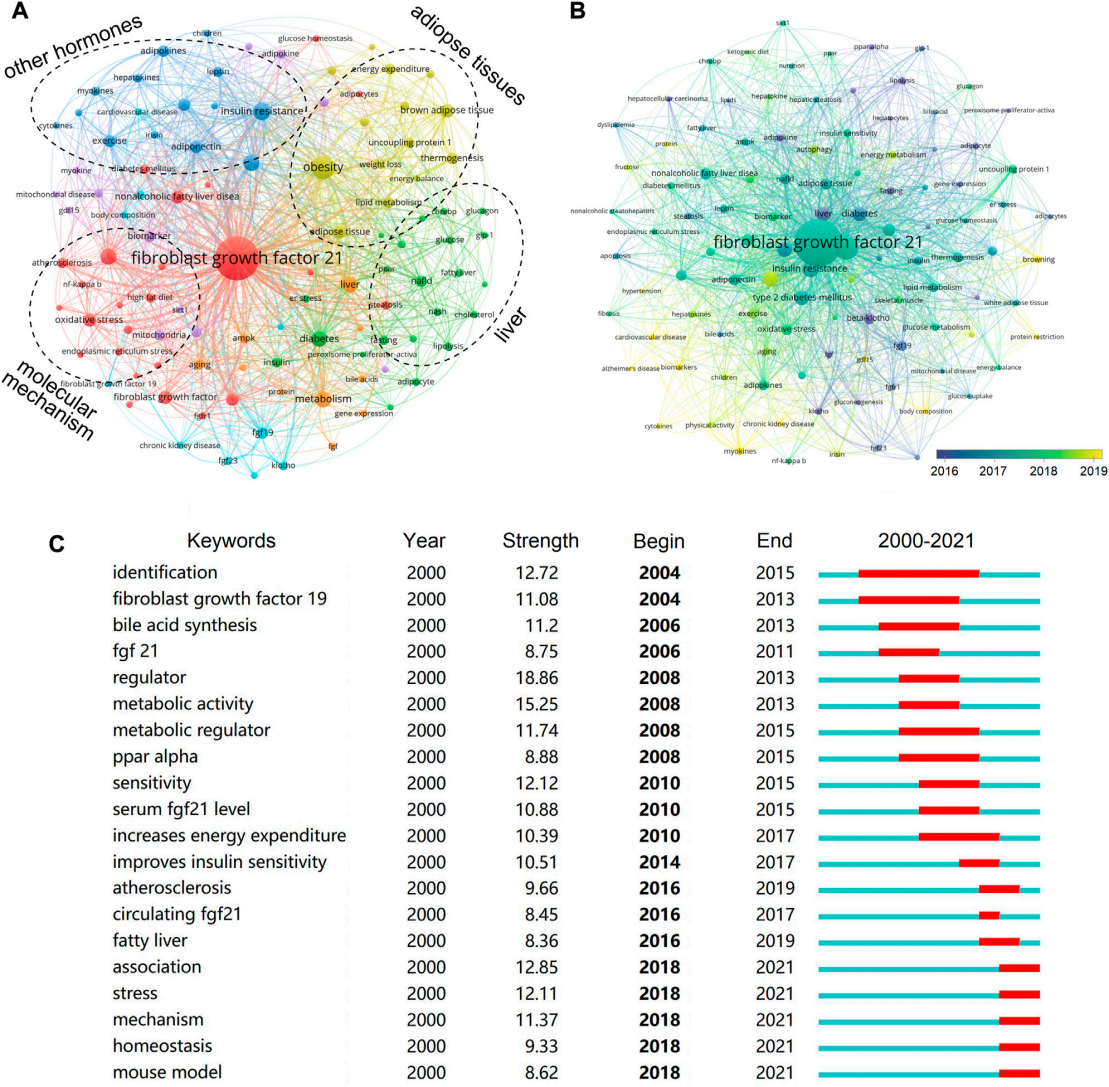
Analysis of keywords in publications. **(A)** Clustering of co-occurrences among keywords. Keywords with close relationships are assigned to one cluster with the same color, and these keywords are roughly divided into four clusters: the association between FGF-21 and other hormones, the action of FGF-21 on adipose tissue, the action of FGF-21 on liver, and the molecular mechanisms of FGF-21. **(B)** Timeline visualization of co-occurrence among keywords. The nodes marked with purple color represent the keywords that appeared relatively earlier, whereas those with yellow color represent the current research focuses. **(C)** Top 20 keywords with the highest citation bursts.

A total of 107 keywords (set as author keywords) were identified as having occurred more than ten times. According to the clustering, the research topic of FGF-21 was roughly divided into four parts, namely, the relationship between FGF-21 and other hormones, the action of FGF-21 on adipose tissues, the action of FGF-21 on liver, and the molecular mechanism of the effects of FGF-21 (Figure 6A), and these parts were the main topics in FGF-21 research. Several novel aspects were found in the co-occurrence network, including “Alzheimer’s disease” (co-occurrence: 10 times), “hypertension” (10), “protein restriction” (11), “body composition” (12), “gdf15” (13), “chronic kidney disease” (14), and “cardiovascular disease” (15), which were all less exposed in FGF-21 research but may have potential significance (Figure 6B). Furthermore, we performed keyword burst detection, and 62 keywords with higher burst strength were extracted. The top 20 keywords are shown in Figure 6C. Consistent with previous results, “identification” and “fibroblast growth factor 19” were major concerns in the initial stage; metabolic effects of FGF-21 were hot topics during the period 2008–2014, and the relationship between FGF-21 and metabolic disease, such as “atherosclerosis” and “fatty liver”, received attention later. In the last four years, “stress”, “mechanism”, “homeostasis”, and “mouse model” were the keywords with higher burst strength (Figure 6C).

### Analysis of publications in the last five years

**FIGURE 7:**
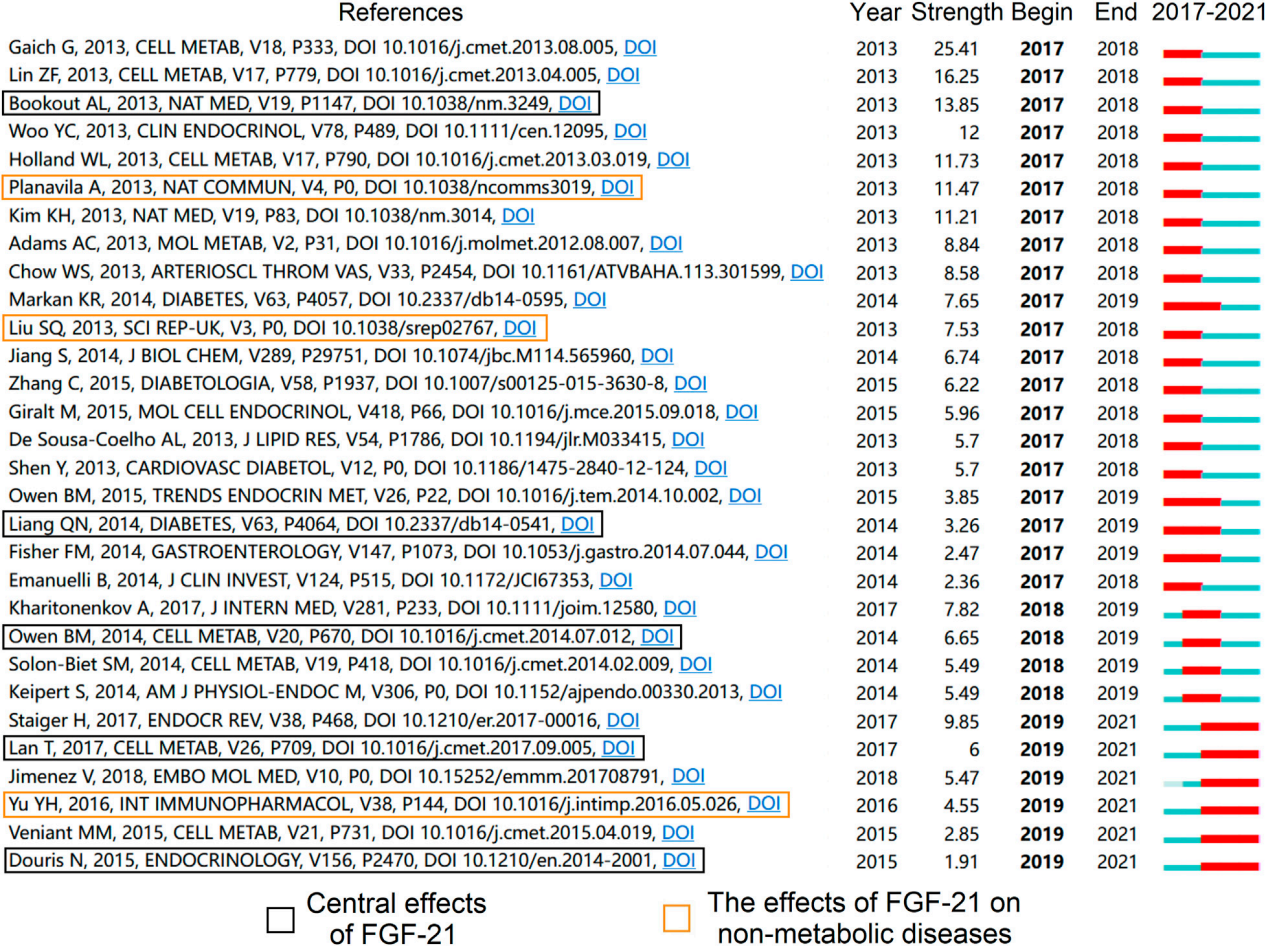
References with the highest citation bursts over the past 5 years (2017–2021).

To further demonstrate the potential directions of FGF-21 research, we analyzed co-cited references in the past five years (2017–2021). Citation burst detection identified 30 references with the strongest citation bursts (Figure 7). Notably, in addition to traditional topics, such as the metabolic effects of FGF-21 on peripheral tissues (Adams et al., 2013b; De Sousa-Coelho et al., 2013; Holland et al., 2013; Kim et al., 2013; Lin et al., 2013; Emanuelli et al., 2014; Fisher et al., 2014; Jiang et al., 2014; Keipert et al., 2014; Markan et al., 2014; Véniant et al., 2015; Zhang et al., 2015; Jimenez et al., 2018) and the association between FGF-21 and metabolic diseases (Chow et al., 2013; Shen et al., 2013), some novel aspects can be found in these publications, namely, the central effects of FGF-21 (Bookout et al., 2013; Liang et al., 2014; Owen et al., 2014; Douris et al., 2015; Lan et al., 2017) and the effects of FGF-21 on non-metabolic diseases (Liu et al., 2013; Planavila et al., 2013; Yu et al., 2016). These studies have extended our understanding of FGF-21 biology, especially the central effects of FGF-21, which may not only be a critical part of future investigations but also a key to comprehending the inconsistencies of the metabolic effects of FGF-21 in human and animal studies (Gaich et al., 2013; Talukdar et al., 2016; Kim et al., 2017; She et al., 2022).

## Discussion

In the present study, we analyzed the main knowledge domain and emerging trends in FGF-21 research through a bibliometric analysis. The results showed that FGF-21 research has entered a mature stage since its rise in 2005, and the United States has always been the leading country in FGF-21-related research studied. Importantly, the clinical translation of FGF-21 remains unclear, especially in glycometabolism, and further investigations on its metabolic functions are still necessary.

### Leading countries in FGF-21 research

Although only three of the top ten productive institutions were from the United States (Supplementary Table S4), eight institutions from the United States demonstrated a higher TLCS in FGF-21 topics (Table 2). In addition, authors from the United States occupied eight of the most influential author list of FGF-21 research, including Alexei Kharitonenkov, Steven A. Kliewer, David J. Mangelsdorf, Moosa Mohammadi, Regina Goetz, Jeffrey S. Flier, Xunshan Ding, and Holly A. Bina (Table 3). Notably, these authors mainly concentrated on physiological research (Steven A. Kliewer, David J. Mangelsdorf, Moosa Mohammadi, Regina Goetz, Jeffrey S. Flier, Xunshan Ding, and Holly A. Bina), translation research (Alexei Kharitonenkov), and drug development (Alexei Kharitonenkov) related to FGF-21. Since physiological research on FGF-21 is the basis of translation and drug development research (Figure 4B), the United States deservedly exhibit a higher influence in FGF-21 research.

It is noteworthy that China published the second highest number of articles in this field (Figure 2A, Table 1, and Supplementary Table S3); nevertheless, the average citation of these articles was relatively low (Table 1; Supplementary Table S3). Correspondingly, the top ten institutions and top ten authors with the highest TLCS only included one Chinese institution (the University of Hong Kong) and one Chinese scholar (Aiming Xu) (Table 2; Table 3). Among the top ten active authors (Supplementary Table S5), Chinese authors mainly concentrated on translational research (Aiming Xu, Xiaokun Li, Desheng Li, Guiping Ren, and Weiping Jia) and drug development research (Desheng Li and Guiping Ren). Thus, Chinese scholars have mainly focused on applications rather than physiological research of FGF-21, which results in a minor impact on FGF-21 research.

### The evolving trends of FGF-21 research

In 2000, Nishimura et al. identified the existence of FGF-21 in mammals (Nishimura et al., 2000); however, its specific function remained unclear for several years. In 2005, Kharitonenkov et al. uncovered the glucose- and lipid-lowering effects of FGF-21. Most importantly, they found that FGF-21 does not induce mitogenesis and hypoglycemia, indicating that FGF-21 is an ideal drug for metabolic diseases (Kharitonenkov et al., 2005). Since then, an increasing number of articles have been published (Figure 1A), and studies on FGF-21 have focused on several aspects, including physiological research, FGF-21-based translational research, and FGF-21-based drug development (Figure 5C). These studies confirmed the anti-diabetic and anti-obesity effects of FGF-21 (Kharitonenkov et al., 2007; Coskun et al., 2008; Xu et al., 2009a), which further accelerated the clinical translation of FGF-21. However, these studies also yielded a series of seemingly contradictory results and challenged the findings of Kharitonenkov et al. (2005). FGF-21 was found to reduce blood glucose during nutrient sufficiency (for example, obesity or during feeding) (Xu et al., 2009a; Berglund et al., 2009; Xu et al., 2009b; Li et al., 2009; Ding et al., 2012; Holland et al., 2013; BonDurant et al., 2017), whereas it increased blood glucose during nutrient-deficient periods (for example, fasting) (Potthoff et al., 2009; Liang et al., 2014). More importantly, FGF-21-based therapy was sufficient to reduce blood glucose in preclinical studies (Adams et al., 2013a; Huang et al., 2013; Kharitonenkov et al., 2013; Kolumam et al., 2015; Weng et al., 2015; Thompson et al., 2016; Baruch et al., 2020), whereas this effect was absent in most of the clinical studies (Gaich et al., 2013; Talukdar et al., 2016; Kim et al., 2017; Baruch et al., 2020). These findings have encouraged researchers to further investigate the physiological roles of FGF-21.

As mentioned earlier, the effects of FGF-21 occur in a metabolic status-dependent manner (Xu et al., 2009a; Berglund et al., 2009; Xu et al., 2009b; Li et al., 2009; Potthoff et al., 2009; Ding et al., 2012; Holland et al., 2013; Liang et al., 2014; BonDurant et al., 2017), suggesting that FGF-21 may act as an important player in maintaining metabolic homeostasis instead of just functioning as an anti-hyperglycemic factor. How does FGF-21 exert different functions under different metabolic conditions? The central nervous system (CNS) senses the metabolic status and coordinates metabolic homeostasis at the whole-body level (Myers and Olson, 2012), and FGF-21 has been shown to cross the blood–brain barrier (Hsuchou et al., 2007). In addition, the hypothalamus, brain stem, and hindbrain express both FGFRs and β-klotho (Bookout et al., 2013; Liang et al., 2014; Von Holstein-Rathlou et al., 2016; Lan et al., 2017; Jensen-Cody et al., 2020), further indicating that FGF-21 may be able to maintain homeostasis through its actions on the CNS. Liang et al. demonstrated that *Fgf21* knockout mice exhibit hypoglycemia during fasting, and further investigations have found that FGF-21 can activate the hypothalamus–pituitary–adrenal (HPA) axis to promote hepatic glucogenesis and elevate glucose levels (Liang et al., 2014). Intracerebroventricular injection of FGF-21 increased sympathetic outflow to adipose tissues, which induced browning of adipose tissues, energy expenditure, and lipolysis (Owen et al., 2014; Douris et al., 2015). Interestingly, the CNS, especially the hypothalamus, responds differently to FGF-21, depending on the metabolic status it detects. Matsui et al. revealed that fasting reduces hypothalamic *Klb* expression, thereby weakening the hypothalamic response to FGF-21 (Matsui et al., 2018). Similarly, FGF-21 can potentiate Ca^2+^ changes in both glucose-excited and glucose-inhibited neurons during the process of glucose increase in cerebrospinal fluid, whereas such effects are absent during the process of glucose decrease (Jensen-Cody et al., 2020). These results suggest that the CNS, especially the hypothalamus, which is a coordinator of whole-body metabolism (Coll and Yeo, 2013), is key to fully understanding the metabolic roles of FGF-21.

**FIGURE 8:**
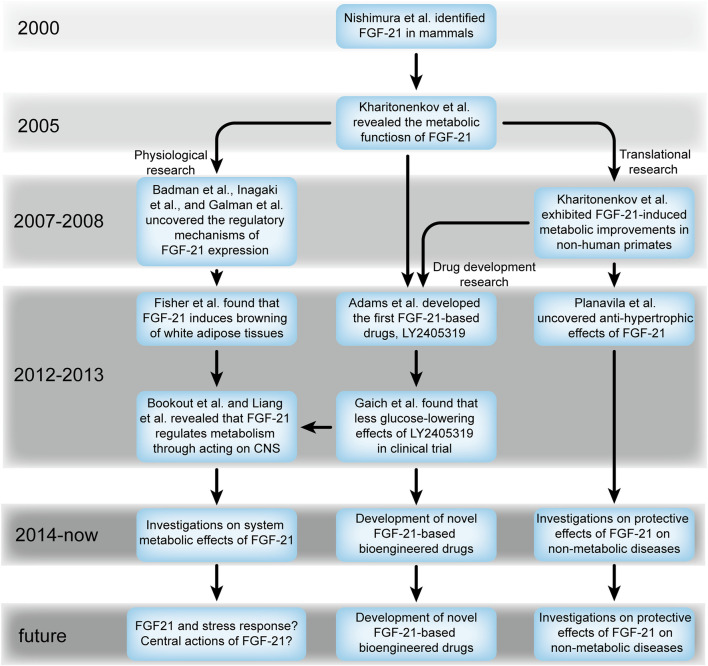
Evolving trends of FGF-21 from 2000 onward.

In addition to metabolic effects, the diagnostic value and non-metabolic effects of FGF-21 have also received attention. Circulating FGF-21 levels were positively correlated with obesity-related metabolic complications (Zhang et al., 2008), renal function (Lin et al., 2011), atherosclerosis (Chow et al., 2013), and hypertension (Semba et al., 2013), indicating that FGF-21 may be used as a biomarker in metabolic or even non-metabolic diseases. Since Planavila et al. uncovered that FGF-21 can suppress cardiac hypertrophy (Planavila et al., 2013), subsequent studies also demonstrated that FGF-21 exerts protective effects against cardiac ischemic injury (Liu et al., 2013) and inflammation (Yu et al., 2016), and FGF-21 was considered a protective factor in non-metabolic diseases. This further extended our understanding of FGF-21 biology (Figure 8).

### Future outlook of FGF-21

Future major directions for FGF-21 research should be based on the current main topics (Figure 8). First, FGF-21 can mitigate metabolic disorders without inducing hypoglycemia or mitogenesis (Kharitonenkov et al., 2005), which shows the future significance of FGF-21-based therapies for metabolic diseases. Thus, the development of novel FGF-21-based bioengineered drugs that overcome the defects of native FGF-21 in clinical applications will remain a hot topic in the near future. Although preclinical findings of FGF-21 translate only partially to clinical trials (Gaich et al., 2013; Talukdar et al., 2016; Kim et al., 2017; Baruch et al., 2020), we speculated that prolonged FGF-21 treatment may be key to realizing its hypoglycemic effects considering the short-term administration of FGF-21 in these studies (Gaich et al., 2013; Talukdar et al., 2016; Kim et al., 2017; Baruch et al., 2020). Indeed, long-term administration of two other FGF-21-based bioengineered drugs, BMS-986036 and AKR-001, significantly reduced blood glucose levels in patients with diabetes (Charles et al., 2019; Harrison et al., 2021). Therefore, studies on novel dosage regimens are required in the future.

The central effects of FGF-21 remain unknown. Although we have obtained some information about the action of FGF-21 on the hypothalamus in recent years, it is inconsiderable. The reports from Jensen-Cody et al. [49] demonstrated that several regions in the CNS express β-klotho, including the suprachiasmatic nucleus, paraventricular nucleus, arcuate nucleus, ventromedial nucleus of the hypothalamus, and area postrema and nucleus tractus solitarius in the hindbrain (Jensen-Cody et al., 2020), and these are the potential acting sites of FGF-21. Thus, these regions are of great significance and may be the key to understanding the metabolic effects of FGF-21 at the whole-body level [for example, the action of FGF-21 on the arcuate nucleus, which is critical for energy sensing, energy expenditure, and food intake (Méndez-Hernández et al., 2020)].

Since FGF-21 was found to exert anti-hypertrophic effects (Planavila et al., 2013), several groups further demonstrated that FGF-21 mitigates several non-metabolic diseases, such as inflammation (Yu et al., 2016), hypertension (Pan et al., 2018), and neurodegeneration (Chen et al., 2019); thus, the non-metabolic effects of FGF-21 are worth exploring, and investigation of its relationship with severe diseases, such as cardiovascular diseases (Planavila et al., 2013; Pan et al., 2018), Alzheimer’s disease (Chen et al., 2019), and chronic kidney disease (Salgado et al., 2021) (Figure 6B), may further extend the understanding and clinical value of FGF-21.

Notably, some studies brought FGF-21 into another wider field. Since Laeger et al. found that protein restriction, which is associated with metabolism and aging (Wang et al., 2022), can induce FGF-21 expression through endoplasmic stress response (Laeger et al., 2014), FGF-21 was considered a stress-related factor and opened a novel aspect to comprehend the metabolic effects of FGF-21. This attracted numerous scholars to investigate the relationship between FGF-21 and protein restriction (Figure 5B), and such an issue will remain a hot topic in the near future due to the complicated association between stress response and metabolic status (Figures 6B and C) (Keipert and Ost, 2021; Lemmer et al., 2021).

### Limitations

Our study had several limitations. First, this study focused on FGF-21, and we enriched the search strategy as much as possible; however, a few studies on FGF-21 were not included in the analysis because some journals were not included in SCIE. Second, the results from VOSviewer and CiteSpace were based on a machine algorithm, which may have caused deviations. Finally, future outlooks were based on the co-occurrence of keywords and citation burst detection, and some potential aspects may not have been included.

## Conclusion

The peripheral metabolic effects of FGF-21, FGF-21-based drug development, and translational research on metabolic diseases are the major topics in this field. Future studies will be conducted on several novel aspects, such as the central metabolic effects of FGF-21 and the effects of FGF-21 on non-metabolic diseases. Notably, the action of FGF-21 on the central nervous system is the key to understanding its integrated metabolic effects at the whole-body level and may constitute the basis of future clinical translational and drug development research.

## Data Availability

The raw data supporting the conclusion of this article will be made available by the authors, without undue reservation.
