# Supplementary material for: Bibliometric analysis of fibroblast growth factor 21 research over the period 2000 to 2021
Source: Front Pharmacol. 2022 Sep 27;13:1011008. doi: 10.3389/fphar.2022.1011008 (PMC9551462; doi:10.3389/fphar.2022.1011008)
Supplement: Supplementary file 1 [file DataSheet1.docx]

**Supplementary Table S1**. Excluded publications

| Type | Title |
| --- | --- |
| Retracted publication | RETRACTED: Increased expression of fibroblast growth factor 21 (FGF21) during chronic undernutrition causes growth hormone insensitivity in chondrocytes by inducing leptin receptor overlapping transcript (LEPROT) and leptin receptor overlapping transcript-like 1 (LEPROTL1) expression. |
|  | RETRACTED: Glucagon and lipid interactions in the regulation of hepatic AMPK signaling and expression of PPAR alpha and FGF21 transcripts in vivo. |

**Supplementary Table S2**. Publication types

| Document Type | Number | TLCS | TGCS |
| --- | --- | --- | --- |
| Article | 2172 | 29497 | 79098 |
| Review | 318 | 2885 | 14884 |

**Supplementary Table S3.** The top 10 productive countries concerning FGF-21

| Rank | Country | Publications | TLCS | TGCS | Average TGCS |
| --- | --- | --- | --- | --- | --- |
| 1 | USA | 923 | 21715 | 54087 | 58.60 |
| 2 | China | 720 | 5328 | 15823 | 21.98 |
| 3 | Japan | 210 | 2443 | 8595 | 40.93 |
| 4 | Germany | 176 | 2550 | 7612 | 43.25 |
| 5 | Spain | 131 | 1258 | 5610 | 42.82 |
| 6 | South Korea | 110 | 812 | 2885 | 26.23 |
| 7 | UK | 110 | 790 | 3523 | 32.03 |
| 8 | Denmark | 83 | 813 | 2298 | 27.69 |
| 9 | Canada | 81 | 390 | 2268 | 28.00 |
| 10 | Italy | 79 | 451 | 3780 | 47.85 |

**Supplementary Table S4.** The top 10 productive institutions concerning FGF-21

| Rank | Institution | Publications | TLCS | TGCS | Average TGCS |
| --- | --- | --- | --- | --- | --- |
| 1 | Wenzhou Medical University | 106 | 745 | 2346 | 22.13 |
| 2 | University of Barcelona | 66 | 731 | 3440 | 52.12 |
| 3 | University of Hong Kong | 59 | 2506 | 4360 | 73.90 |
| 4 | Harvard Medical School | 57 | 272 | 2051 | 35.98 |
| 5 | University of Copenhagen | 57 | 629 | 1908 | 33.47 |
| 6 | Shanghai Jiao Tong University | 54 | 750 | 1723 | 31.91 |
| 7 | Northeast Agriculture University | 53 | 426 | 897 | 16.92 |
| 8 | Harvard University | 46 | 2482 | 5485 | 119.24 |
| 9 | Kyoto University | 42 | 1486 | 3769 | 89.74 |
| 10 | Amgen Incorporation | 40 | 1673 | 3025 | 75.63 |

**Supplementary Table S5.** The top 10 active authors concerning FGF-21

| Rank | Name | Publications | TLCS | TGCS | Institutions |
| --- | --- | --- | --- | --- | --- |
| 1 | Xiaokun Li | 68 | 888 | 2396 | Wenzhou Medical University |
| 2 | Kharitonenkov Alexei | 64 | 6990 | 11085 | AK Biotechnology LLC |
| 3 | Francesc Villarroya | 49 | 722 | 3044 | Universitat de Barcelona |
| 4 | Deshan Li | 47 | 1401 | 2275 | Northeast Agricultural University |
| 5 | Aimin Xu | 44 | 2417 | 4078 | The University of Hong Kong |
| 6 | Andrew C. Adams | 39 | 1238 | 2639 | Lilly Research Laboratories |
| 7 | Marta Giralt | 34 | 659 | 2398 | Universitat de Barcelona |
| 8 | Nobuyuki Itoh | 33 | 1417 | 3506 | Kyoto University |
| 9 | Guiping Ren | 32 | 353 | 651 | Northeast Agricultural University |
| 10 | Weiping Jia | 29 | 625 | 1370 | Shanghai Jiaotong University |

**Supplementary Table S6.** The top 10 journals concerning FGF-21

| Rank | Journal | Counts | Impact factor (2021) | TLCS | TGCS | H index |
| --- | --- | --- | --- | --- | --- | --- |
| 1 | PLoS One | 91 | 3.752 | 0 | 3599 | 38 |
| 2 | Scientific Reports | 81 | 4.996 | 0 | 1398 | 22 |
| 3 | Molecular Metabolism | 53 | 8.586 | 888 | 1889 | 23 |
| 4 | Endocrinology | 51 | 5.051 | 2641 | 4394 | 30 |
| 5 | Cell Metabolism | 43 | 31.373 | 4428 | 9002 | 39 |
| 6 | Diabetes | 43 | 9.337 | 2918 | 5001 | 29 |
| 7 | International Journal of Molecular Sciences | 40 | 6.208 | 3 | 679 | 14 |
| 8 | Journal of Clinical Endocrinology & Metabolism | 40 | 6.134 | 907 | 1539 | 25 |
| 9 | Biochemical and Biophysical Research Communications | 36 | 3.322 | 417 | 1171 | 14 |
| 10 | Journal of Biological Chemistry | 36 | 5.486 | 1216 | 2779 | 26 |
